# Protective Effects and Possible Mechanisms of Actions of Bushen Cuyun Recipe on Diminished Ovarian Reserve Induced by Cyclophosphamide in Rats

**DOI:** 10.3389/fphar.2020.00546

**Published:** 2020-05-13

**Authors:** Mei Jiang, Weiling Wang, Jingxuan Zhang, Chunguo Wang, Yucong Bi, Pin Li, Song Yang, Jialin Li, Yan-Tong Xu, Ting Wang

**Affiliations:** ^1^Beijing Research Institute of Chinese Medicine, Beijing University of Chinese Medicine, Beijing, China; ^2^School of Life Science, Beijing University of Chinese Medicine, Beijing, China

**Keywords:** Bushen Cuyun recipe, diminished ovarian reserve, network pharmacology, HPOA, pyroptosis

## Abstract

**Backgrounds:**

Diminished ovarian reserve (DOR) contributes significantly to female infertility. Bushen Cuyun Recipe (BCR, Tradename Yueliang Yin), a product marketed in China, has shown effects in the treatment of female infertility in clinical practices of traditional Chinese medicine (TCM). In this study, we aimed to investigate the chemical compositions of BCR and its efficacy based on scientific evidence and pharmacological mechanisms in DOR treatments.

**Methods:**

The chemical compositions of BCR were determined by the UHPLC-LTQ-Orbitrap MS method. DOR was induced in a rat model by intraperitoneal injection of cyclophosphamide (CTX) 90 mg/kg once. After the CTX treatment for 14 days, rats were intragastrically administrated deionized water, dehydroepiandrosterone (DHEA), or BCR in low, middle, and high doses for 30 days. Ovarian index, ovarian morphology, follicle number, and anti-Müllerian hormone (AMH) in serum were determined to assess the effects of BCR. To investigate possible action mechanisms, network pharmacological analysis was used to predict possible pathways in the effects of BCR on female infertility. In experimental studies, the contents of hormones in the hypothalamic-pituitary-ovarian axis (HPOA, including estradiol (E_2_), follicle-stimulating hormone (FSH), and gonadotropin-releasing hormone (GnRH)) and pyroptosis-related proteins, including gasdermin D (GSDMD), caspase-1, and interleukin-18 (IL-18), in ovarian were detected by ELISA, immunofluorescence and Western blot.

**Results:**

Chemical studies revealed a total 84 components in BCR, which included 43 flavonoids, 13 triterpenoids, 11 phenolic acids, 8 alkaloids, 1 coumarin, 1 anthraquinone, and 7 other components. After treatments with BCR, the ovarian morphology, ovarian index, estrous cycle, growing follicles and corpus luteum from last ovulation, and serum AMH in DOR rats were significantly improved. Network pharmacological analysis suggested that the NOD-like receptor signaling pathway ranked No. 1 among the mechanisms by which BCR affects female infertility. Experimental results demonstrated that the content of serum FSH in DOR rats was significantly decreased and the contents of serum GnRH and E_2_ were significantly elevated after BCR treatment and that the elevated level of GSDMD, caspase-1, and IL-18 was significantly reversed in BCR-treated rats.

**Conclusions:**

The chemical compositions of BCR were first identified in the present study. BCR was demonstrated to show protective effects on DOR. The possible mechanisms of BCR on DOR might be mediated by regulating gonadal hormones of the HPOA and protecting granulosa cells in ovary against pyroptosis.

## Introduction

Diminished ovarian reserve (DOR) refers to reproductive-age women with regular menses whose response to ovarian stimulation or fecundity is reduced compared with healthy women of comparable age (Practice Committee of the American Society for Reproductive, 2015). The prevalence of DOR increased from 19% to 26% between 2004 and 2011, so it represents a major challenge in reproductive medicine (Devine et al., 2015). As a complex disease, DOR endangers women’s physical and mental health. It is well known that pregnancy depends on normal follicles and endometrial receptivity. Dysfunction of reproductive endocrine or improper use of clinical ovulation-promoting drugs could result in a lack of synchronization between follicles and endometrium development and can finally lead to infertility (Shahine et al., 2016).

The etiology and pathogenesis of DOR remain unclear so far, but it has been reported that DOR may be related to age (Fan et al., 2019), immunity (Silva et al., 2014), heredity, environment, radiotherapy and chemotherapy (Smith et al., 2013), surgery, psychological stress (Nicoloro-SantaBarbara et al., 2017), and infectious factors (Soylu Karapinar et al., 2017). A prospective cohort study found that poor ovarian response was related to age and apoptosis of ovarian granulosa cells (Fan et al., 2019). Hyperfunction of the autoimmune system might damage ovary through antigen-antibody reactions (Silva et al., 2014). Clinical studies found that chemical substances such as parabens might be related to DOR (Smith et al., 2013). Long-term anxiety could cause dysfunction of the hypothalamic-pituitary-ovarian axis (HPOA), which can lead to DOR or premature ovarian failure (Nicoloro-SantaBarbara et al., 2017). Among these factors, alkylating chemotherapy agents, such as cyclophosphamide (CTX), which is highly gonad-toxic, led to a decrease in ovarian function and in anti-Müllerian hormone (AMH) (Ben-Aharon et al., 2015). It could induce ovarian damage by activation of the PI3K/AKT and mTOR pathways (Goldman et al., 2017), leading to primordial follicle activation and follicular “burnout” (Chang et al., 2015). Recently, it is reported that pyroptosis occurs in ovarian granular cells when ovary is damaged (Wang and Wang, 2018; Wang et al., 2019). This suggests that pyroptosis of ovarian granular cells may lead to the decline of ovarian function and that inhibition of the pyroptosis of ovarian granular cells may be a potential therapy for DOR.

Current treatments for DOR include hormone replacement therapy (HRT), ovulation induction, and assisted fertility treatment (AFT). There have been debates on the risk-benefit balance in HRT for decades. Early observations showed that it increased risks of coronary heart disease (CHD) and breast cancer. However, newer research showed that CHD and mortality are reduced when HRT is initiated soon after menopause (Lobo, 2017). Currently, it is generally accepted that HRT significantly decreases the incidence of various symptoms and improves quality of life. In younger healthy women, the risk–benefit balance is positive for using HRT. Nevertheless, HRT has strict indications and contraindications, which require that patients return regularly hospital so that the risks of treatment can be evaluated, and this limits its use in clinical applications (Panel, 2017). For ovulation induction, the first-line drug in clinical practices is clomiphene citrate, which causes thin endometrium as well as high ovulation and low pregnancy occurrence (Bjelica et al., 2016; Reed et al., 2018). Letrozole, an aromatase inhibitor traditionally used for breast cancer, is also used for ovulation induction. Although it does not result in thin endometrium, it has shown potent side effects such as dizziness, gastrointestinal upset, and flu (Brueggemeier, 2006). Gonadotrophin-releasing hormone antagonists (GnRH-A) treatment could suppress the expression and activity of luteinizing hormone (LH) receptors in DOR patients (Pinski et al., 1996), and studies have reported that it reduces the pregnancy rate by decreasing endometrial receptivity and disturbing decidualization during implantation (Wu et al., 2012). AFT refers to in vitro fertilization and embryo transfer, etc., which is beyond drug treatments. So far, treatments for DOR are leaving needs unmet, and it is necessary to develop new therapies for DOR treatment. There is a long history of clinical treatments for DOR in traditional Chinese medicine (TCM). TCM treatments emphasize holistic regulation of ovary functions. TCM can synchronize the development of follicles and endometrium (Jiang et al., 2019). Thus, TCM provides an opportunity to develop novel drugs for DOR infertility (Xin et al., 2018). Bushen Cuyun Recipe (BCR) (Tradename Yueling Yin) is a marketed product in China with which positive effects on female infertility have been observed, with few side effects. However, the chemical compositions of BCR and its efficacy and pharmacological mechanisms in DOR have remained unknown.

Animal models of DOR are an important tool for studying the efficacy and pharmacological mechanisms of BCR. So far, animal models of DOR infertility include chemical poison models, such as 4-vinylcyclohexene diepoxide (Hassa et al., 2015), chemotherapy drug models, such as cyclophosphamide (CTX) (Meirow et al., 2004), stress models, such as the chronic unpredictable stress paradigm (Gao et al., 2019), autoimmune models, such as zona pellucida 3 fragments (Wang et al., 2018), and natural aging models (Shen et al., 2017). For instance, a rat model of DOR infertility was induced by 4-vinylcyclohexene diepoxide (Hassa et al., 2015), cyclophosphamide was used to set up a mouse model of DOR infertility (Meirow et al., 2004), and an AC57BL/6 mouse model of DOR infertility was established *via* an 8-week chronic unpredictable stress paradigm (Gao et al., 2019). Among these models, those induced by chemotherapies, such as CTX, showed evident advantages: (1) pathological alterations in the DOR model were similar to clinical observations in patients, (2) pathological alterations in DOR model could be reversed by drugs, (3) the method was simple and feasible, and (4) the results of this DOR model proved to be reproducible (Dehghani et al., 2018). Therefore, the DOR model induced by CTX was used in this study.

## Materials and Methods

### Chemicals and Reagents

The chemicals and reagents used were the standard substances kaempferol, quercetin, hesperetin, and hesperidin (National Institutes for Food and Drug Control), Cyclophosphamide (Baxter Oncology GmbH, Halle, Germany; No. 8K274A), Dehydroepiandrosterone (General Nutrition Corporation Pittsburgh, PA, USA; No. 61411H18), Rat FSH enzyme-linked immunosorbent assay (ELISA) kit (Cloud-Clone Corp. Wuhan, China; No. CEA830Ra), Rat AMH ELISA kit (Cloud-Clone Corp. Wuhan, China; No. CEA228Ra), Rat GnRH ELISA kit (Cloud-Clone Corp. Wuhan, China; No. CEA843Ra), Rat E_2_ ELISA kit (Cloud-Clone Corp. Wuhan, China; No. CEA461Ge), TUNEL apoptosis assay kit (KeyGENBio TECH. Jiangsu, China; No. KGA 7072), 4′, 6-diamidino-2-phenylindole (DAPI) staining kit (KeyGENBio TECH. Jiangsu, China; No. KGA 215-10), Normal sheep serum (ZSGB-BIO. Beijing, China; No. ZLI-9022), Triton X-100 (KeyGENBio TECH. Jiangsu, China; No. KGF011), Caspase-1 rabbit polyclonal antibody (Proteintech Group, Inc. Chicago, USA; No. 22915-1-AP), GAPDH mouse monoclonal antibody (Proteintech Group, Inc. Chicago, USA; No. 60004-1-lg), Anti-GSDMD antibody (Abcam Cambridge, MA, USA; No. ab219800), Rat IL-18 ELISA kit (RayBiotech, Inc. Georgia, USA; No. P97636), and CoraLite594-conjugated donkey anti-rabbit IgG (H+L) (Proteintech Group, Inc. Chicago, USA; No. SA00013-8).

### Preparation of Bushen Cuyun Recipe (BCR) samples

BCR comprises 10 herbs: 14.5% Polygon atumsibiricum Red., 14.5% Discorea opposita L., 14.5% Poria cocos (Schw.) Wolf, 10.9% Lycium barbarum L., 10.9% Morus alba L., 10.9% Rubus chingii Hu, 4.3% Cinnamomum cassia (L.) J.Presl, 4.3% Foeniculum vulgare Mill., 4.3% Syzygium aromaticum (L.) Merr. & L.M.Perry, and 10.9% Citrus × aurantium L. The herbs were provided by Chongqing JuqiNuomei Pharmaceutical Co., Ltd. (Chongqing, China) and identified by Prof. Xiangri Li, Beijing University of Chinese Medicine. The samples (No. 171101) were deposited in the Beijing Research Institute of Chinese Medicine, Beijing University of Chinese Medicine.

Preparation of BCR samples was as follows: 3.4 kg of the crude BCR drug was boiled twice for 2 hours each time with 10 times (w/v) distilled H_2_O. The combined extraction was filtered and then concentrated at 80°C under reduced pressure for 6 hrs. After vacuum drying, 1.0 kg of dry powder was obtained, with a yield ratio of 29.2%. As body surface area (BSA) scaling has been recommended by the U.S. Food and Drug Administration for converting the dose of a test drug from animal species to human clinical trials (Rockville et al., 2005; Nair and Jacob, 2016), we calculated the test doses of BCR in animals based on the BSA ratio compared with human. The details were as follows. The dose of BCR in terms of raw materials for an adult human is 22.99 g/day. The body weight of an adult human was calculated as 70 kg. Based on the extract ratio of 29.2% of dry powder/raw materials of BCR, the dose of dry powder for adult human was 6.71 g/day. To calculate the dose for animals, the ratio of equivalent dose based on surface area converted between rat and human was 6. The low dose is half of the equivalent clinical dose, while the high dose is double the equivalent clinical dose. The positive drug was administrated to animals at the equivalent dose of human clinical dose. Accordingly, the three doses of BCR were 0.3, 0.6, and 1.2 g/kg/d, and the DHEA group was 6mg/kg/d.

### Determination of Components of BCR by the UHPLC-LTQ-Orbitrap MS Method

The chemical components of BCR were determined by the ultra-high-pressure liquid chromatography coupled with linear ion trap/electrostatic field orbital trap tandem high-resolution mass spectrometry (UHPLC-LTQ-Orbitrap MS) method. UPLC analysis was performed on a DionexUtimate 3000 UHPLC Plus Focused Ultra High-Performance Liquid Chromatography System (Thermo Scientific, Santa Clara, CA, USA). A 20.0 ml volume of 50% methanol was added 2.0 g (precisely weighed) of BCR powder, and the mixture was weighed, ultrasonically treated for 40 minutes using an ultrasonic cleaning instrument (KQ-500DB CNC, Kunshan ultrasonic instrument Co., Ltd., Kunshan, Jiangsu, China), then weighed again. The lost weight was supplemented by 50% methanol, and the filtrate was taken through 0.45 μm microporous membrane. The standards such as kaempferol, quercetin, hesperetin, hesperidin, and caffeic acid were accurately weighed, dissolved in methanol with a standard solution of 1 mg/ml in concentration, and filtered through 0.45 μm microporous membrane. Samples or strands were separated on an AQUITY UPLC C_18_ column (2.1 mm*100 mm, 1.7μm) at 35°C. The mobile phase consisted of 0.1% formic acid aqueous solution (A) and acetonitrile solution (B). The gradient elution conditions were as follows: 0-3 min (5%-5% B), 3-45 min (5%-75% B), 45-45.1 min (75-5% B), 45.1-50 min (5%-5% B). The flow rate was 0.3 ml/min, and the injection volume was 2.0 μl. ESI-MS analyses were performed on an LTQ-Oribitrap XL Linear ion trap tandem electrostatic field orbital trap mass spectrometer (Thermo Scientific, Santa Clara, CA, USA). Samples of BCR were detected in positive ion detection mode, and the spray and capillary voltages were set to 4.0 KV and 35.0 V, respectively. The tube lens voltage was 110 V, and the source temperature was set to 350°C. Nitrogen (purity > 99.99%) was used as both the sheath gas (40 arb) and auxiliary gas (20 arb). Then samples were analyzed in negative ion detection mode, with the spray and capillary voltages set to 3.0 KV and 35.0 V, respectively. The tube lens was set to 110 V, and the source temperature was set to 350°C. Nitrogen (purity > 99.99%) was used as both the sheath gas (30 arb) and auxiliary gas (10 arb).

Data-dependent acquisition (ddms3) of high-resolution Fourier transform (TF, full scan, resolution 30000) and CID fragmentation were used for positive and negative ion data acquisition. The compositions of BCR were confirmed by referring to the retention time of each chemical component, high-resolution precise molecular weight, and MSn multi-level fragment information detected by LC-MS and combined with the extraction of ion flow map and standard product information and related literature.

### Pathway Analysis of BCR Based on Network Pharmacology

Compound Data Preparation and ADME Screening: The corresponding herb targets were obtained by conducting a search in the traditional Chinese medicine systems pharmacology database (TCMSP, Version: 2.3 http://tcmspw.com/tcmsp.php) and PubMed-reported literatures. Afterward, two important *in silico* ADME indexes, including OB (oral bioavailability) and DL (drug-likeness), were employed to screen the candidate active ingredients. The threshold values of these two indexes were set as OB≥30% and DL≥0.18, respectively (Pang et al., 2018). These ingredients, which met the two criteria above, were selected as candidate molecules for further analysis.

Target Fishing and Network Construction: DOR was not encoded by Medical Subject Headings (MeSH), so female infertility (MeSH^®^ID: D007247) was used for searching disease targets. The targets were searched in the Comparative Toxicogenomics Database (CTD, Data updated 2019 http://ctdbase.org/). The matched herb targets and disease targets were regarded as the predicted targets of BCR. Then the predicted targets were uploaded to the Search Tool for the Retrieval of Interacting Genes/Proteins database (STRING, Version 11 https://string-db.org/) for GO enrichment analysis and KEGG pathway analysis. KEGG pathway analysis was viewed as a bubble chart on the OmicShare website (http://www.omicshare.com/).

### Animals

Total 54 SPF female Sprague-Dawley rats, 8 weeks, were purchased from Beijing Vital River Laboratory Animal Technique Co., Ltd. (Beijing, China, certificate No. SCXK [Beijing] 2016-0006). All animals were housed in groups with *ad libitum* food and water. The holding room was maintained at room temperature at 22 ± 2 °C with humid conditions (45%–55%) and a 12 h light/day cycle. This study was approved by the Ethics Committee for Animal Care and Treatment of Beijing University of Chinese Medicine (BUCM-4-2019061001-2039).

### DOR Model Induced by CTX

A DOR rat model was established as described by Khedr (2015), with minor modifications. Rats used for DOR experiments were screened by estrous cycle *via* vaginal smear examination. The normal estrous cycle of rat is 4–5 days. There were 54 rats with normal estrous cycle, and these rats were randomly assigned into six groups: control, DOR, low (L-BCR), middle (M-BCR), high (H-BCR), and DHEA (9 each). According to our preliminary experiments, a dose of CTX 90 mg/kg was used to induce DOR in rats. CTX was dissolved in saline. All rats in the DOR, BCR, and DHEA groups received CTX intraperitoneal (ip) injection once, while rats in the control group received an equal volume of saline. After CTX administration for 14 days, the DOR model was ready to be used. The animals were treated with BCR or DHEA for 30 days by intragastric administration. The control and DOR rats were administrated an equal volume of deionized water.

### Test of Estrous Cycle

Vaginal smears were undertaken daily for 30 days after BCR and DHEA treatments. A flexible plastic dropper with purified water was used to obtain vaginal exfoliated cells of rats every morning at 9 am. The collected solution was smeared on a slide and was then examined under a microscope (EVOS FL Auto 2; Thermo Fisher; Carlsbad, CA). The stage of estrous cycle was determined as follows (Barker et al., 2018): proestrus (nucleated epithelial cells dominant), estrous (cornified epithelial cells dominant), metestrus (similar proportion of nucleated epithelial cells, cornified epithelial cells, and leukocytes), and diestrus (leukocytes dominant).

### Calculation of Ovarian Index and Count of Follicle

Rats were sacrificed after treatments and ovaries were harvested. The ovarian index was calculated as follows:

$$\mathrm{ovarian}\;\mathrm{index}(\%)=\frac{\text{ovarian weight }(g)}{\text{body weight }(g)}\times 100\%$$

The ovaries were fixed in 4% paraformaldehyde for 48 h, embedded with paraffin, and then serial-sectioned with a thickness of 3 μm. The ovary section that was largest in diameter in the different groups was stained with hematoxylin-eosin (H&E), and follicular counts were performed. The different follicles were classified according to the following characteristics: primordial follicle: monolayer and flattened granulose cells around the oocytes; primary follicle: a single layer of cubic granulosa cells around the oocytes; secondary follicle: multi-layer and cuboidal granulosa cells surrounding the oocytes; antral follicle: follicular antrum-cumulus oophorus-complex (Pedersen and Peters, 1968; Plowchalk et al., 1992). Primary follicles, secondary follicles, and antral follicles were collectively called growth follicles. After ovulation, the granulosa cells of the follicle remnant undergo hypertrophy and hyperplasia. This process is called luteinization and results in mature corpus luteum (Westwood, 2008). Corpus luteum from the last ovulation was large, and the H&E staining characteristics started changing from basophilic to eosinophilic. Corpus luteum from the last ovulation indicates the ovulation condition to some degree (Dixon et al., 2014). In this study, the numbers of primordial follicles, growth follicles, and corpus lutea from the last ovulation in the current estrous cycle were counted.

### Immunofluorescence TUNEL Staining of Ovarian Tissue

To test DNA damage of the granulosa cells in the ovarian tissues, TUNEL staining was undertaken according to the manufacturer’s instructions. Briefly, sections containing ovarian stroma were incubated with proteinase K for 30 min at 37°C, followed by another incubation with biotin-11-dUTP and TdT Enzyme (mixed with 1:4) reagents for 1 h at 37°C. Then, streptavidin-Fluorescein and Labeling Buffer (mixed with 1:9) reagents were dropped to cover the section, and it was incubated for 30 min at 37 °C without light. The nuclear staining was dyed with DAPI. The sections were observed under a fluorescence microscope. Green fluorescence staining in the cells was considered positive for nuclear DNA fragmentation.

### Immunofluorescence Staining for Caspase-1 of Ovarian Tissue

Ovary sections were fixed in 4% paraformaldehyde for 15 min, and non-speciﬁc bindings were blocked by blocking solution containing 10% normal goat serum and 0.5% Triton X-100 for 1 h at room temperature. Ovary tissue sections were then incubated with Caspase-1 (Proteintech, No.22915-1-AP, 1:400) overnight at 4 °C. Secondary antibody (CoraLite594-conjugated donkey anti-rabbit IgG, Proteintech, No.SA00013-8, 1:400) was added for 1 h at room temperature without light. Finally, nuclear staining was performed using DAPI, and slides were evaluated under a fluorescent microscope. Red fluorescence staining in the cells was considered positive expression for caspase-1.

### Enzyme-Linked Immunosorbent Assay

Serum concentrations of Gonadotropin-releasing hormone (GnRH), follicle-stimulating hormone (FSH), anti-Müllerian hormone (AMH), estradiol (E_2_), and ovarian interleukin-18 (IL-18) were detected by enzyme-linked immunosorbent assay (ELISA). All procedures followed the instructions of the kits.

### Western Blot Analysis

In brief, tissue proteins were extracted in RIPA lysis buffer (C1053; Applygen Technologies Inc.; Beijing, China) containing protease inhibitor (P1265; Applygen Technologies Inc.; Beijing, China) and protein phosphatase inhibitor mixture (P1260; Applygen Technologies Inc.; Beijing, China). The quantification of proteins was measured by BCA protein assay kit (Beyotime Biotechnology Inc.; Shanghai, China). Equal amounts of proteins were separated by 10% SDS-PAGE and transferred to PVDF membranes (Millipore, Billerica, MA) then incubated overnight at 4°C with antibodies against GSDMD (abcam, No.ab219800, 1:1000), caspase-1 (Proteintech, No.22915-1-AP, 1:500), and GAPDH (Proteintech, No.60004-1-lg, 1:5000). The goat-anti-rabbit IgG-HRP (Proteintech, No.SA00001-2, 1:10000) secondary antibody was used. Enhanced chemiluminescence was used for color development. The gray values of the protein bands were analyzed by Image J, and the relative protein expression was calculated using GAPDH as the internal standard.

### Statistical Analysis

Statistical analysis was conducted using Kruskal-Wallis tests. The results were presented as mean ± SEM. Statistical significance is indicated as *p* < 0.05 or *p <* 0.01.

## Results

### Chemical Compositions of BCR

**Figure 1:**
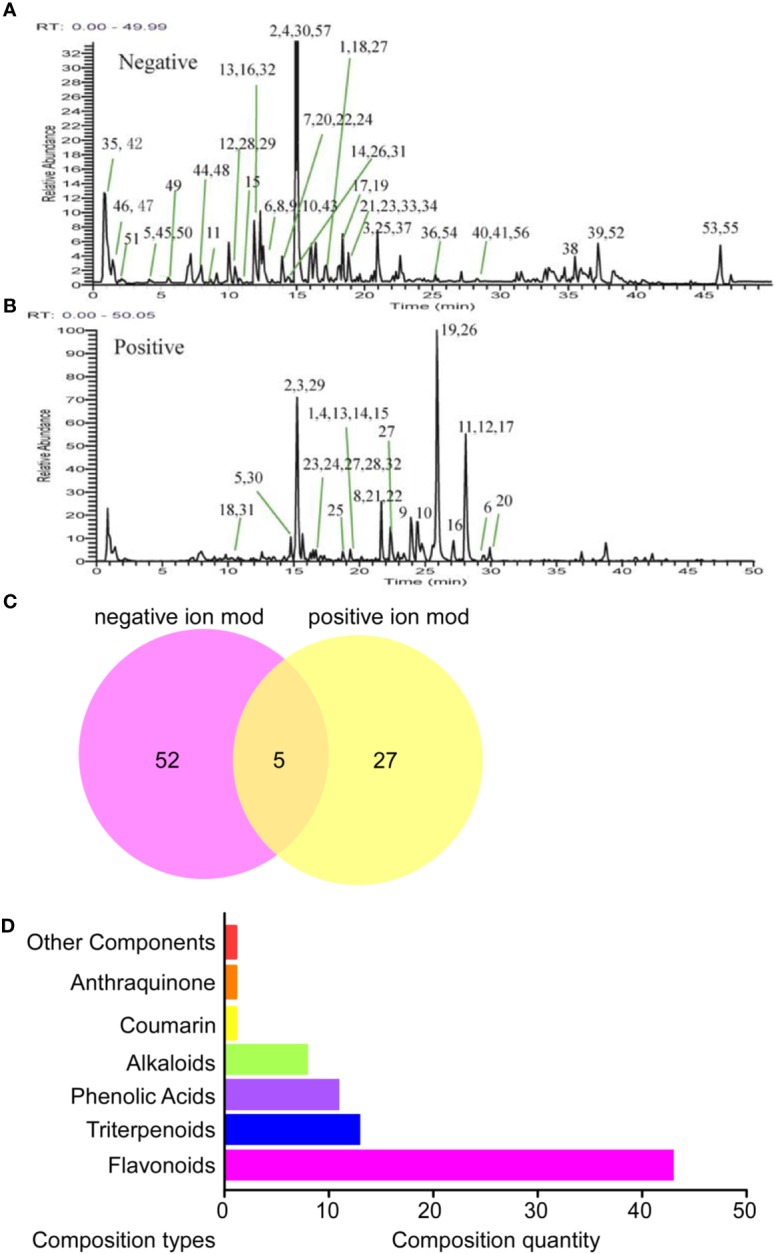
Identification results of the main chemical components of BCR by LTQ-Orbitrap MS. **(A)** Total ion flow diagram of BCR in positive ion mode; **(B)** Total ion flow diagram of BCR in anion mode; **(C)** Number of chemical components in BCR identified by positive and negative ion models; **(D)** Identification of the main components in BCR by LTQ-Orbitrap MS.

A total of 84 components were identified in BCR, including 32 components in the positive ion mode, 57 components in the negative ion mode, and 5 components in the positive and negative ion mode (**Figures 1A–C**). The identified 84 chemical components included 43 flavonoids, 13 triterpenoids, 11 phenolic acids, 8 alkaloids, 1 coumarin, 1 anthraquinone, and 7 other components (**Figure 1D**), among which 5 chemical components were identified by comparison to standard components: kaempferol, quercetin, hesperetin, hesperidin, and caffeic acid.

### BCR Increased Ovarian Index and Improved Estrous Cycle

**Figure 2:**
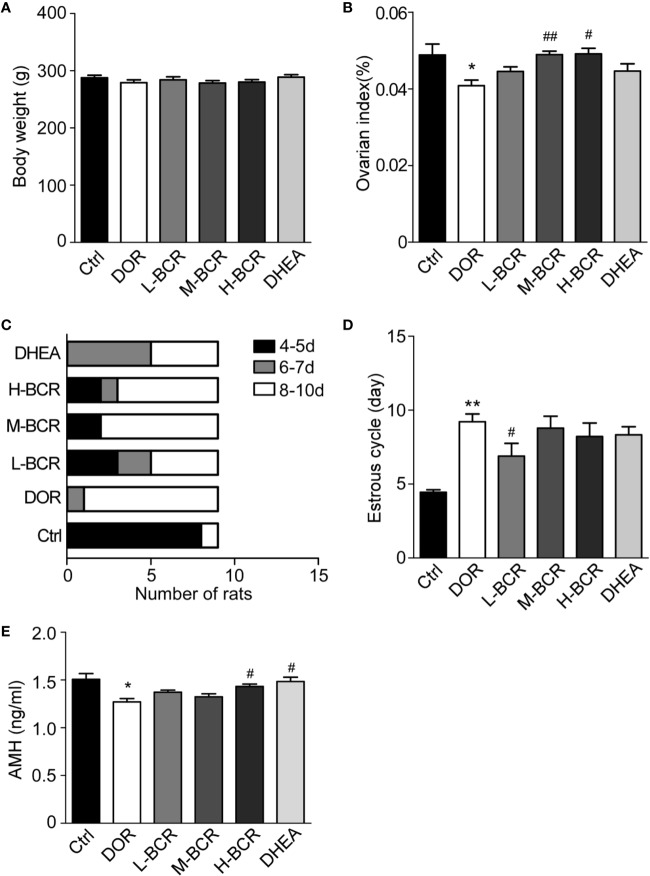
Cyclophosphamide (CTX)-induced decline in ovarian reserve (DOR) rats was reflected by decreased ovarian index, a prolonged estrous cycle, and decreased serum AMH, and BCR treatments reversed the changes in DOR rats. **(A)** Body weight. **(B)** Ovarian index. **(C)** Constituent ratios of the estrous cycle. **(D)** Average length of estrous cycle. Values are represented as means ± SEM, n=9 per group. **P < 0.01 versus control group; ^#^*P*c< 0.05, ^##^*P* < 0.01 versus DOR group.

As shown in **Figure 2A**, there were no significant differences in body weight among the groups. As indicated in **Figure 2B**, the ovarian index of the DOR group was significantly decreased compared with the control group (*p* < 0.05), while it was significantly increased in the M-BCR (*p* < 0.01) and H-BCR (*p* < 0.05) groups compared with the DOR group.

The estrous cycle of rats was continuously measured to evaluate the protective effects of BCR on ovary. The results showed that the estrous cycle of the DOR group was irregular and was prolonged to 8–10 days. In the L-BCR group, about 30.00% of rats returned to the original estrus cycle, and 22.22% showed a shorter estrous cycle in than the DOR group, ranging from 6 to 7 days (**Figures 2C, D**). The estrous cycle of rats in the M-BCR and H-BCR groups was also shorter than in DOR rats. Additionally, about half of the rats treated with DHEA showed a shorter estrous cycle, ranging from 6 to 7 days (**Figures 2C, D**).

### BCR Elevated Content of Serum AMH

AMH is one of the most important indexes of ovarian reserve in the clinical diagnosis of DOR. In this experiment, the serum AMH of rats in the DOR group was significantly decreased compared to that in the control group (*p* < 0.05) (**Figure 2E**). Serum AMH was increased in H-BCR and DHEA groups compared with the DOR group (*p* < 0.05) (**Figure 2E**).

### BCR Relieved Reduction in Numbers of Growing Follicles and Corpus Lutea From Last Ovulation

**Figure 3:**
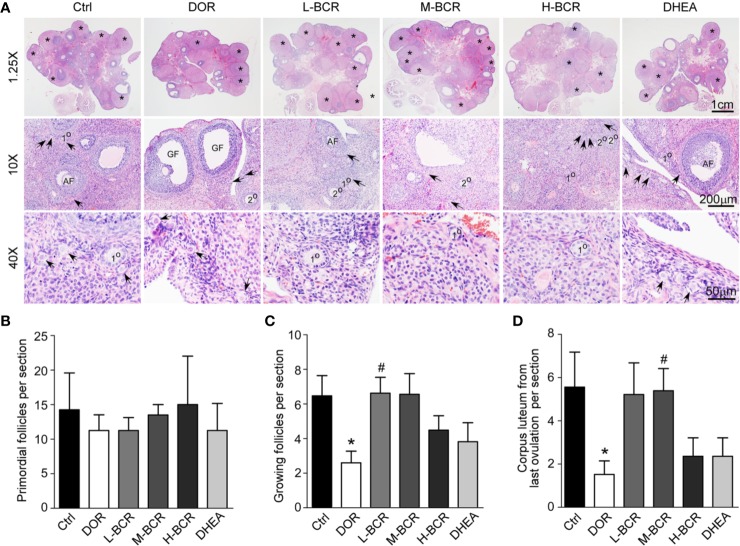
BCR administration improved morphological alterations of ovaries in DOR, including increased numbers of growing follicles and corpus lutea from last ovulation. **(A)** HE staining of ovaries with 1.25× (top line), 10× (middle line), and 40× (bottom line) magnification. Asterisks indicated corpus lutea from last ovulation. Arrows indicate primordial follicles. 1° indicates primary follicles. 2° indicates secondary follicles. AF indicates antral follicles. Primary follicles, secondary follicles, and antral follicles were considered to be growing follicles. GF indicates mature Graafian follicles. **(B)** Number of primordial follicles. **(C)** Number of growing follicles. **(D)** Number of corpus lutea from last ovulation. Values are represented as mean ± SEM, n = 5-6. *P < 0.05 versus control group; ^#^*P* < 0.05 versus DOR group.

To evaluate the effects of BCR on the DOR model on the basis of growing follicles and corpus lutea, ovaries of rats were stained with the HE (**Figure 3A**). The number of primordial follicles was similar among all groups (**Figures 3A, B**). The number of growing follicles significantly decreased in the DOR group (*p* < 0.05) (**Figures 3A, C**). The number of growing follicles in the L-BCR (*p* < 0.05) group was significantly increased compared to DOR rats. Corpus lutea from the last ovulation were significantly decreased in number in DOR rats compared to in the control group (*p* < 0.05), and the number of corpus lutea from the last ovulation was significantly elevated by M-BCR (*p* < 0.05) compared with the DOR group (**Figures 3A, D**).

### BCR Regulated Homeostasis of the Hypothalamic-Pituitary-Ovarian Axis (HPOA)

**Figure 4:**
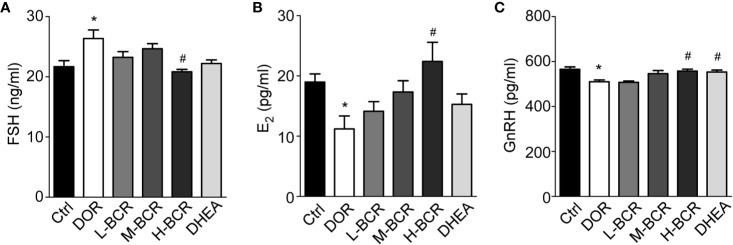
BCR administration improved dysregulated hypothalamic-pituitary-ovarian axis (HPOA) in DOR rats. **(A)** Serum level of FSH. **(B)** Serum level of E_2_. **(C)** Serum level of GnRH. Values are represented as mean ± SEM, n = 6 per group. *P < 0.05 versus control group; ^#^*P* < 0.05 versus DOR group.

To investigate the effects of BCR on HPOA, the serum FSH, E_2_, and GnRH contents were detected. Serum FSH (*p* < 0.05) were significantly increased, while serum E_2_ (*p* < 0.05) and GnRH (*p* < 0.05) were significantly decreased in DOR rats compared with control group rats (**Figure 4A**). After BCR treatments, serum FSH was significantly decreased in H-BCR (*p* < 0.05) compared with the DOR group. Serum E_2_ was significantly increased in H-BCR compared with the DOR group in a dose-dependent manner (**Figure 4B**). Serum GnRH was significantly increased in the H-BCR (*p* < 0.05) and DHEA (*p* < 0.05) groups compared with the DOR group (**Figure 4C**).

### Analysis of Possible Mechanisms of BCR in Female Infertility Through Network Pharmacology

**Figure 5:**
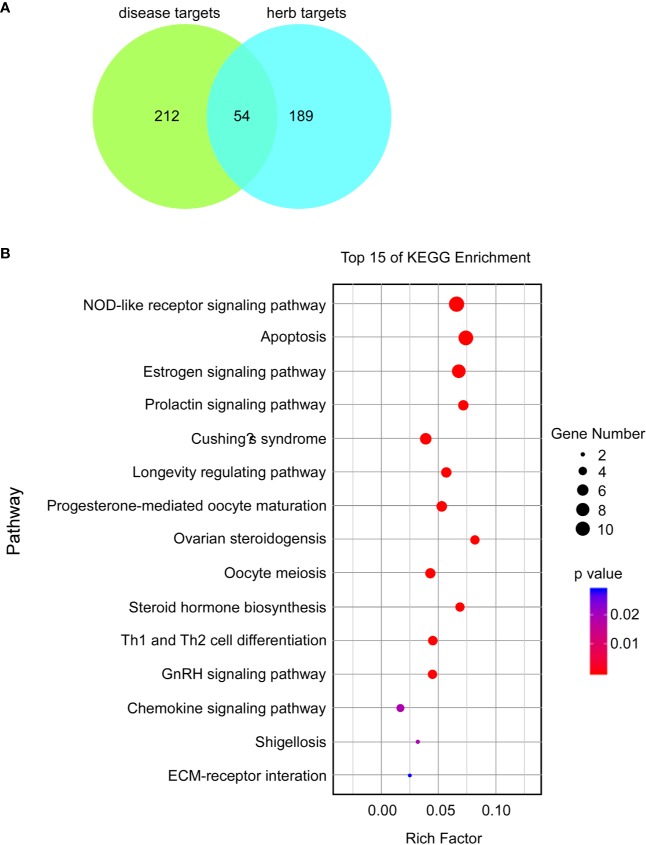
Potential proteins and possible mechanisms of BCR in the treatment of female infertility based on analysis of KEGG enrichment. **(A)** Venn graph of predicted targets. **(B)** Bubble chart of top 15 KEGG enrichments; a lower p-value indicates a more related pathway.

A total of 266 disease targets of female infertility and 243 herb targets of BCR were obtained, and 54 predicted targets and 15 related pathways were constructed by protein-protein interaction (PPI) network (**Figure 5**). The pathways were ranked as follows: NOD-like receptor signaling pathway, apoptosis, estrogen signaling pathway, prolactin signaling pathway, Cushing’s syndrome, longevity-regulating pathway, progesterone-mediated oocyte maturation, ovarian steroidogenesis, oocyte meiosis, steroid hormone biosynthesis, Th1 and Th2 cell differentiation, GnRH signaling pathway, chemokine signaling pathway, shigellosis, and ECM-receptor interaction (**Figure 5B**).

**Figure 6:**
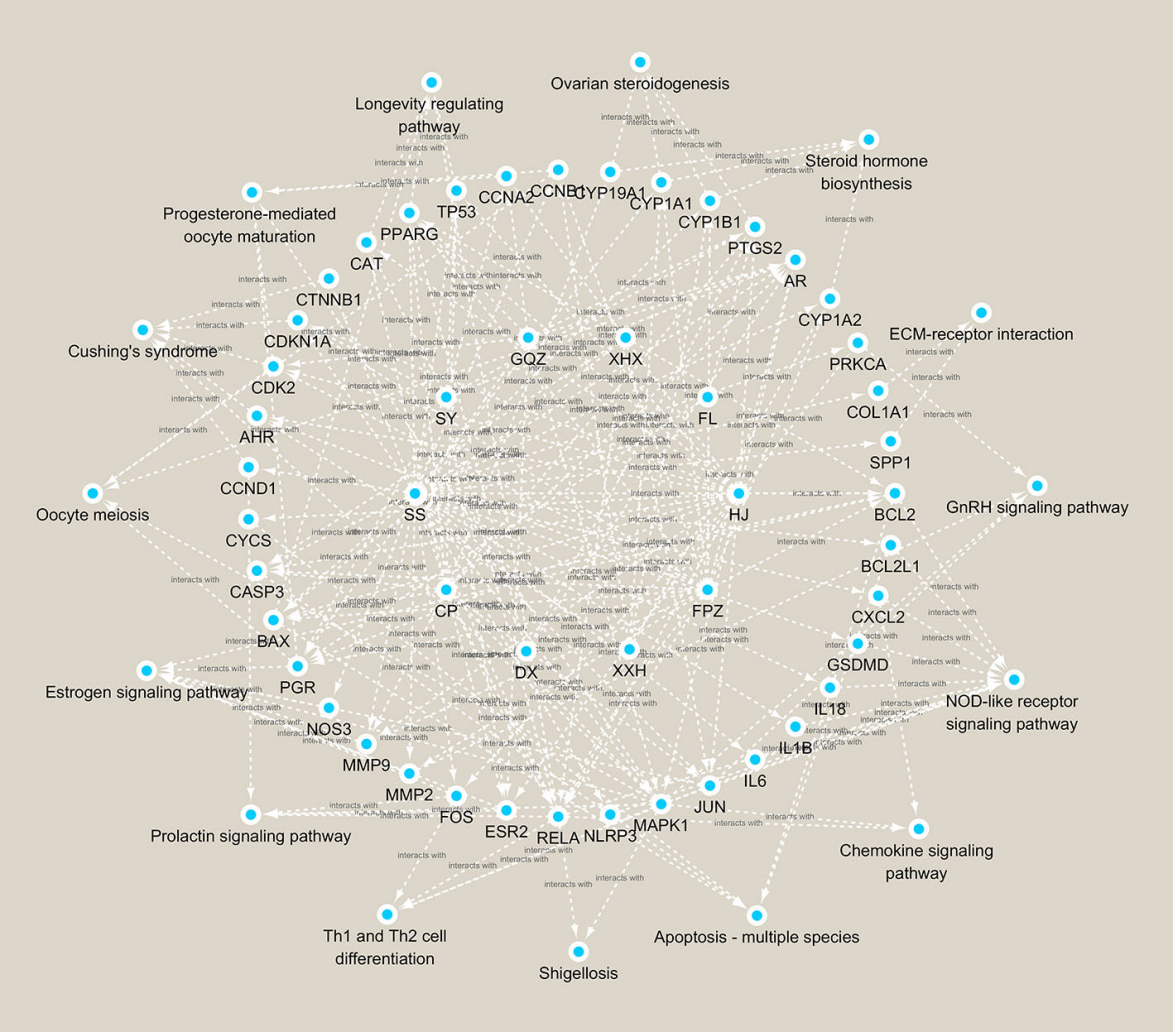
Compound-Target-Pathway network of active ingredients in BCR for infertility disease.

The basic principles of traditional Chinese medicine were established on the theory that multiple components of herbs could interact with multiple targets of diseases. In the present study, network analysis was performed to predict the possible network of BCR in treating infertility. According to the calculation principles of point–point and point–line, we put compounds, targets, and pathways into a network diagram, and predicted the importance according to “degree, closeness, and betweenness.” A “Compound-Target-Pathway network of active ingredients in BCR for infertility disease” network was constructed (**Figure 6**). Based on the sequence of importance, the herbs in BCR were ranked as follows: HJ (*Polygon atumsibiricum* Red.), SY (*Discorea opposita* L.), FL (*Poria cocos* (Schw.) Wolf), GQZ (*Lycium barbarum* L.), SS (*Morus alba* L.), FPZ (*Rubus chingii* Hu), RG (*Cinnamomum cassia* (L.) J.Presl), XHX (*Foeniculum vulgare* Mill.), DX (*Syzygium aromaticum* (L.) Merr. & L.M.Perry), and CP (*Citrus × aurantium* L.). The Compound-Target-Pathway network showed that a herb could interact with multiple components, and a component could also interact with several targets related to female infertility (**Table 1**). This demonstrated that the therapeutic effects of BCR were mediated by compositions of BCR interacting with female infertility related targets by a “multi-target and multi-pathway” mode. The big data computing model predicted that NOD-like receptor signaling pathway was ranked as No.1 in the treatment pathways of BCR for DOR. The results of network pharmacology pointed out that BCR may regulate BCL2, BCL2L1, CXCL2, Gasdermin D (GSDMD), IL18, IL1B, IL6, JUN, MAPK1, NLRP3, and RELA, which are the key points of the NOD-like receptor signaling pathway. Among these, GSDMD, IL18, IL1B, and NPLR3 proved to be the key targets of pyroptosis. Based on the above results, we proposed that BCR might regulate GSDMD, IL18, IL1B, and NPLR3 to act on the pyroptosis pathway and carried on the exploration verification in the animal model.

**Table 1:** Candidate pathways and targets of BCR for DOR.

KEGG pathway	Target	KEGG pathway	Target
NOD-like receptor signaling pathway	BCL2	Longevity-regulating pathway	BAX
	BCL2L1		CAT
	CXCL2		PPARG
	GSDMD		RELA
	IL18		TP53
	IL1B		
	IL6	Ovarian steroidogenesis	CYP19A1
	JUN		CYP1A1
	MAPK1		CYP1B1
	NLRP3		PTGS2
	RELA		
		Oocyte meiosis	AR
Estrogen signaling pathway	BCL2		CCNB1
	ESR2		CDK2
	FOS		MAPK1
	JUN		PGR
	MAPK1		
	MMP2	Steroid hormone biosynthesis	CYP19A1
	MMP9		CYP1A1
	NOS3		CYP1A2
	PGR		CYP1B1
Apoptosis - multiple species	BAX	Th1 and Th2 cell differentiation	FOS
	BCL2		JUN
	BCL2L1		MAPK1
	CASP3		RELA
	CYCS		
		GnRH signaling pathway	JUN
Prolactin signaling pathway	CCND1		MAPK1
	ESR2		MMP2
	FOS		PRKCA
	MAPK1		
	RELA	Chemokine signaling pathway	CXCL2
			MAPK1
Cushing’s syndrome	AHR		RELA
	CCND1		
	CDK2	Shigellosis	MAPK1
	CDKN1A		RELA
	CTNNB1		
	MAPK1	ECM-receptor interaction	COL1A1
			SPP1
Progesterone-mediated oocyte maturation	CCNA2		
	CCNB1		
	CDK2		
	MAPK1		
	PGR		

### BCR Attenuated Pyroptosis of Ovarian Granulosa Cells in DOR Rats

**Figure 7:**
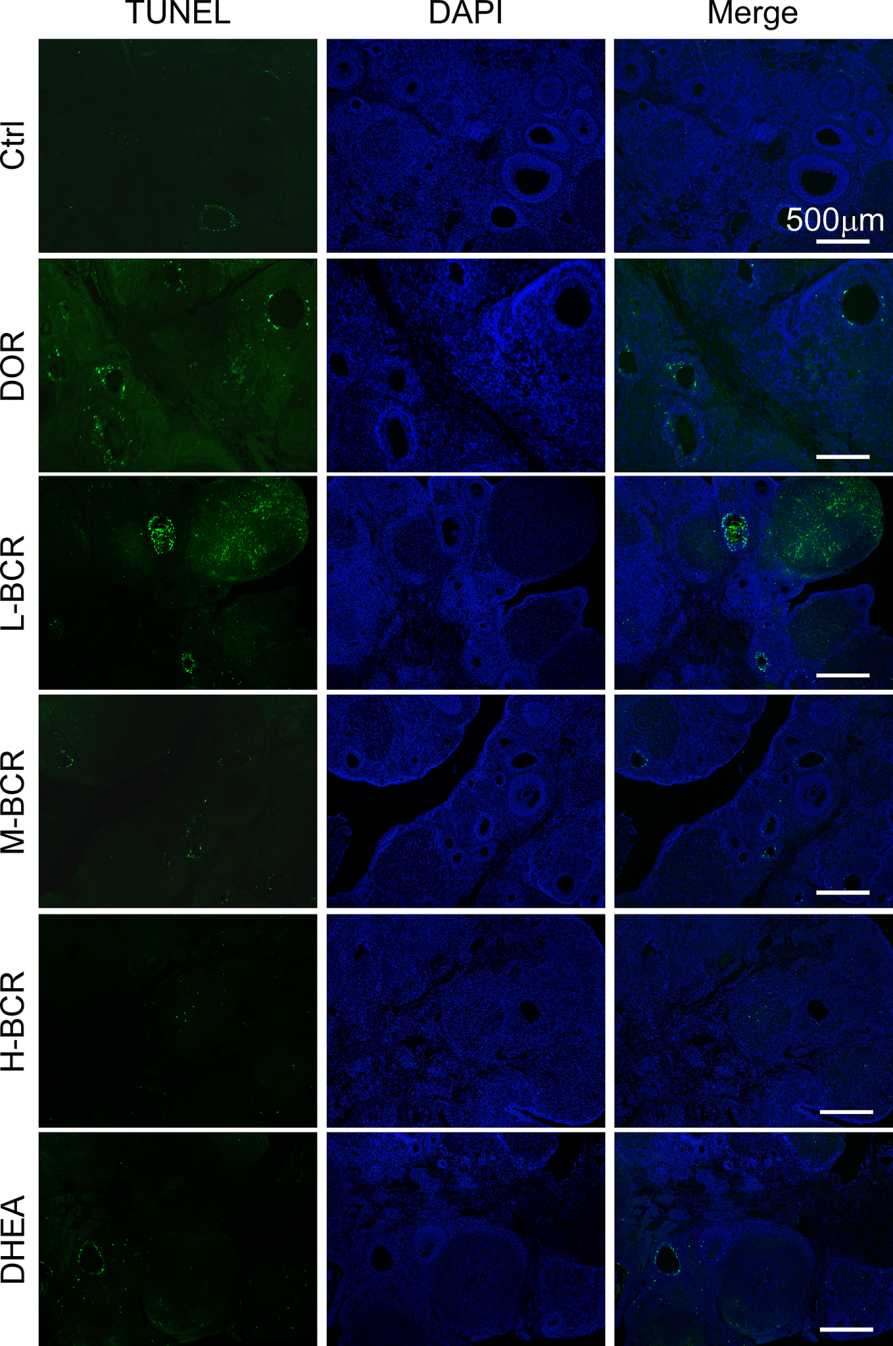
BCR administration reduced apoptosis of granulosa cells in ovaries of DOR rats. Immunofluorescence staining for damaged DNA (green) and DAPI (blue). Scalebar = 500 μm.

**Figure 8:**
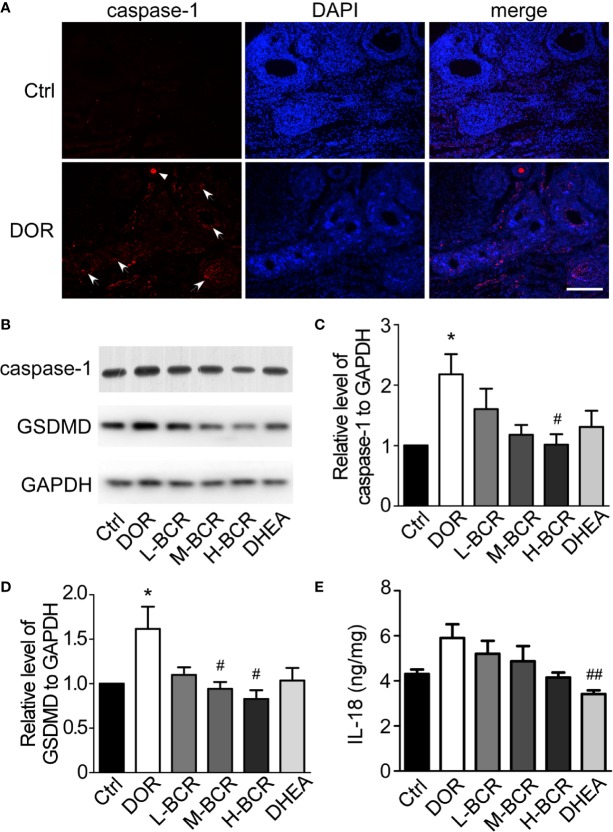
BCR administration attenuated pyroptosis of granulosa cells in ovaries of DOR rats. **(A)** Immunofluorescence staining for caspase-1 (red) and DAPI (blue). Scale bar = 200 μm. Arrows indicate caspase-1-positive follicles. Triangle indicates caspase-1-positive oocyte. **(B)** Representative Western blot image of expression of GSDMD, caspase-1, and GAPDH. **(C)** Gray value ratio of caspase-1 to GAPDH. **(D)** Gray value ratio of GSDMD to GAPDH. **(E)** Ratio of IL-18 to total protein in ovaries by ELISA. Values are represented as mean ± SEM, n = 5–6 per group. *P < 0.05 versus control group; ^#^*P* < 0.05, ^##^*P* < 0.01 versus DOR group.

Immunofluorescence staining of TUNEL revealed that CTX destroyed ovarian follicles by inducing DNA damage in granulosa cells (**Figure 7**). Next, pyroptosis in ovaries was detected by immunofluorescence staining for caspase-1. The positive expression of caspase-1 was observed in follicles of the ovaries in DOR rats (**Figure 8A**). Furthermore, as shown in **Figures 8B, C**, the ovarian expression of caspase-1 was 2-fold higher in the DOR group than in the control group (*p* < 0.05). Additionally, the levels of gasdermin D (GSDMD), which is downstream of caspase-1, were 1.5-fold higher in the DOR group than that in the control group (*p* < 0.05) (**Figures 8B, D, E**). After treatment, the expressions of caspase-1, GSDMD, and IL-18 were decreased compared with the DOR group in the groups with different dosages of BCR in a dose-dependent manner: significant difference in caspase-1 expression was shown in the H-BCR group (*p* < 0.05, **Figures 8B, C**), and the expression of GSDMD was significantly decreased in the M- (*p* < 0.05) and H-BCR (*p* < 0.05) groups (**Figures 8B, D**). Meanwhile, the elevated level of IL-18 was reversed in the H-BCR and DHEA groups, but the difference was only significant for the DHEA group (*p* < 0.01, **Figure 8E**).

## Discussion

The chemical compositions of BCR were first determined by the UHPLC-LTQ-Orbitrap MS method in the present study. The results showed that flavonoids, triterpenoids, phenolic acids, and alkaloids were the main components of BCR. Since few studies were yet available on the bioactivities of triterpenoids, phenolic acids, and alkaloids in the treatment of infertility, this suggested that flavonoids played key roles in BCR treatments on DOR. According to previous reports, flavonoids were demonstrated to show evident bioactivities in DOR treatments. For instance, total flavonoids regulated serum levels of FSH, luteinizing hormone (LH), and testosterone (T) in polycystic ovary syndrome rats by inhibiting the JAK2/STAT3 pathway (Zhou et al., 2019). Flavonoids also inhibited oxidative stress of endometrial cells to improve pregnancy outcome (Estany et al., 2007). The flavonoids in BCR included hesperetin, hesperidin, kaempferol, quercetin, rutin, etc. It was reported that hesperetin, hesperidin, and quercetin protected ovary from injury through antioxidative and anti-apoptosis effects (Cakir Gungor et al., 2014; Khedr, 2015; Naseer et al., 2017; Nna et al., 2017; Aizawa et al., 2018; Khadrawy et al., 2019; Rashidi et al., 2019; Sirotkin et al., 2019a; Sirotkin et al., 2019b). Kaempferol and quercetin had estrogen-like activities and regulated serum hormones (Yang et al., 2018; Jafari Khorchani et al., 2019; Neisy et al., 2019). Hesperetin protected rat ovarian ischemia-reperfusion injury by alleviating tissue apoptosis (Cakir Gungor et al., 2014). Hesperidin promoted ovarian protection against damage resulting from CTX chemotherapy, possibly by inhibiting oxidative stress of ovarian granulosa cells (Khedr, 2015; Aizawa et al., 2018). Quercetin inhibited the oxidative stress of granulosa cells (Khadrawy et al., 2019; Rashidi et al., 2019), reduced the apoptosis of granulosa cells or oocytes (Naseer et al., 2017; Nna et al., 2017; Sirotkin et al., 2019a; Sirotkin et al., 2019b), and improved the inflammatory microcirculation of ovary (Wang et al., 2017). Meanwhile, quercetin was a phytoestrogen and had a structure and oestrogenic activity similar to 17β-oestradiol (Yang et al., 2018), and it promoted E_2_ and LH secretion in uterus and ovary (Jafari Khorchani et al., 2019; Neisy et al., 2019). Kaempferol promoted primordial follicle activation and cell proliferation through the PI3K/AKT pathway (Santos et al., 2019). In addition, kaempferol could regulate the level of serum estrogen and progesterone, resume the estrous cycle, and increase the weight of uterus in ovariectomized rats, which supported the estrogenic potency of kaempferol (Swar et al., 2017). Rutin could ameliorate ovarian injury in rats through its antioxidative effect (Lins et al., 2017; Nayki et al., 2018) and up-regulate the levels of ovarian steroidogenic enzymes (Hu et al., 2017). The above evidences provide valuable clues on flavonoids for further studies on the possible action mechanisms of BCR in the treatment of DOR.

This is the first study demonstrating BCR showed effects on DOR on the basis of scientific evidences. The effects of BCR on ovarian functions were evaluated in the following aspects. It is known that the estrous cycle and ovarian corpus lutea from the last ovulation can reflect the status of ovulation of rats (Abedel-Majed et al., 2019; Liman and Ates, 2020). The growing follicle count, and especially the antral follicle count, is a reliable method for predicting the total number of mature follicles and pregnancy rate (Keane et al., 2017). In the present study, it was demonstrated that the estrous cycle was significantly decreased and that the ovarian index and the numbers of ovarian corpus lutea from the last ovulation and of growing follicles were significantly increased after BCR treatments compared with DOR rats. This result suggested that BCR significantly improved the ovulation of rats. AMH is a member of the transforming growth factor β-super family produced by ovarian granulosa cells in female. The serum AMH content alters with developmental phase, starting from puberty and disappearing in the menopause period. As an indicator of ovarian reserve function, AMH showed obvious advantages. Alteration of AMH appears earlier than changes of FSH, E_2_, inhibin B, and antral follicle count in reflecting the decline in ovarian reserve function with aging, and its content is not influenced by menstrual cycle, hormone contraceptives, and pregnancy (Sefrioui et al., 2019). It is the most accurate biomarker of ovarian aging. In this study, CTX decreased serum AMH content, however, H-BCR treatments significantly increased it. This suggested that BCR could improve ovarian reserve by protecting ovarian granulosa cells. This evidence demonstrated the protective effects from CTX damage of BCR on ovarian follicles.

A DOR model induced by CTX was used in the present study. CTX can destroy the storage of primordial follicles, so that it increases risk of premature ovarian failure (POF) or DOR, and can seriously affect fertility in women of reproductive age (Bellusci et al., 2019; Spears et al., 2019). Accordingly, this side effect of CTX was used to establish a DOR model in rats. In our preliminary experiments, different doses of CTX (50mg/kg, 75mg/kg, and 90mg/kg) were tested. The results indicated that CTX 90 mg/kg could induce significant and consistent alterations in the estrous cycle and serum FSH. Moreover, CTX has been widely used in chemotherapy for cancers. Evident side effects exist in CTX treatment in cancer patients, in particular, serious reproductive toxicity. The protective effects of BCR on DOR suggested that BCR could be used in cancer patients treated by CTX to relieve reproductive toxicity.

DHEA was used as positive control in the present study. The reasons were: first, DHEA is an essential prohormone for ovarian follicular steroidogenesis. Although it shows weak androgen activity, DHEA is a precursor for the synthesis of both testosterone and estrogen. It can therefore directly or indirectly enhance expression of FSH receptor in ovary, improving the sensitivity of ovarian granulosa cells to gonadotropins, increasing the number of antral follicles, reducing the chromosomal abnormality of embryos, and improving the quantity and quality of oocytes and embryos (Nagels et al., 2015; Qin et al., 2017). Second, studies showed that DHEA improved ovarian function in poor responders, reduced follicular atresia, and increased number of active oocytes (Mahmoud et al., 2018; Schwarze et al., 2018). It evidently increased the peak estradiol level in women with DOR in clinical observations (Casson et al., 2000). Finally, DHEA was used as a positive drug to improve ovarian function in many reported animal studies (Mahmoud et al., 2018; Sozen et al., 2019).

Results of network pharmacology analysis suggested that the possible mechanisms of BCR in the treatment of female infertility were multi-target and multi-pathway. The highly related pathways including the estrogen signaling pathway, prolactin signaling pathway, progesterone-mediated oocyte maturation, ovarian steroidogenesis, oocyte meiosis, steroid hormone biosynthesis, and the GnRH signaling pathway, as well as proteins, especially estrogen receptor β (ESR2), progesterone receptor (PGR), Gasdermin D (GSDMD), NPLR3, interleukin-1β (IL-1B), interleukin-18 (IL-18), apoptosis regulator Bcl-2(BCL2), Bcl-2-like protein 1 (BCL2L1), caspase-3 (CASP3), interleukin-6 (IL-6), transcription factor AP-1 (JUN), and mitogen-activated protein kinase 1 (MAPK1), which were all related to HPOA activities and programmed cell death (such as pyroptosis and apoptosis) (**Figures 5** and **6**). In addition, the potential proteins aforementioned such as GSDMD, NPLR3, IL-1β, and IL-18 are involved in the NOD-like receptor signaling pathway, which play important roles in pyroptosis (**Figure 5B**). The analysis based on network pharmacology shed light on a novel strategy for research into BCR. Therefore, to investigate the possible action mechanisms of BCR in DOR, homeostasis of the HPOA and pyroptosis of ovarian granulosa cells were studied.

It is well known that HPOA activities involve a multiple-level feedback system to maintain homeostasis of neuroendocrinological regulation in reproductive functions. As is known, FSH stimulates follicular growth and maturation. It binds to FSHR on granulosa cells to activate aromatase and promote the production of E_2_. E_2_ plays an important role in the maintenance of follicles and can cooperate with FSH to promote follicular development (Shi et al., 2019). Meanwhile, E_2_ has negative feedback effects on FSH *via* HPOA (Liu et al., 2014). Our results showed that CTX led to DNA damage and pyroptosis of granulosa cells, so it resulted in a fall in E_2_. The fall in E_2_ elevated serum FSH *via* negative feedback mechanisms, which deceased GnRH in the same way. These results were consistent with previous studies (Huang et al., 2019; Zheng et al., 2019). BCR treatments decreased the FSH level and increased the GnRH level in DOR rats. Moreover, after BCR treatments, the estrous cycle was significantly shortened and ovulation was promoted, so BCR caused significant improvements in ovarian reserve function.

Besides the protective effects of BCR on ovarian follicles and HPOA activities, BCR could protect against pyroptosis of ovarian granulosa cells in DOR rats (**Figure 8**). Previous reports found that CTX disrupted the ultrastructure of granulosa cells and induced apoptosis and autophagy, finally resulting in ovarian failure (Liu et al., 2016). Recent research found that CTX also induced pyroptosis, mainly manifested in DNA damage (Chen et al., 2016). Pyroptosis, caspase-1-dependent cell death is a new form of programmed cell death (Miao et al., 2011). Caspase-1 is activated by nucleotide-binding domain leucine-rich repeat-containing proteins NLRP under stimulations of bacteria, viruses, and toxic foreign substances. It causes pro-caspase-1 to be processed into two cleaved subunits and leads to extracellular release of pro-inflammatory cytokines, including interleukin-1β (IL-1β) and IL-18 (Wu et al., 2015). Gasdermin D (GSDMD) is the executor of cell scorch. It is cut by caspase-1/11/4/5 to form N-terminal and C-terminal. N-terminal anchors lipids and other components on cell membrane assemble into oligomers (Rogers et al., 2019), which allows mature IL-1β and IL-18 molecules to pass through (Feng et al., 2018). These pores also cause cations, especially Ca^2+^, to flow in. These cations destroy cell osmotic pressure and electrochemical gradient and finally cause cell scorching (Katsnelson et al., 2015). In this experiment, TUNEL assay showed that there was apoptosis in the ovarian granulosa cells induced by CTX, which was consistent with previous reports (Liu et al., 2016). Immunofluorescence assay of caspase-1 showed that CTX induced ovarian granulosa cell pyroptosis. Further experiments revealed that the expressions of GSDMD, caspase-1, and IL-18 were significantly increased in DOR rats induced by CTX. These results confirmed that pyroptosis existed in ovary of DOR rat and played an important role in ovarian function. It was found that pyroptosis inhibitors could significantly improve the level of E_2_ and the density of transplanted follicles in an ovarian transplantation experiment in mice, suggesting that inhibiting pyroptosis could ameliorate ovarian function (Wang et al., 2019). Compared with DOR rats, the expressions of GSDMD, caspase-1, and IL-18 were significantly decreased after BCR treatment. This suggested that BCR improved ovarian function by decreasing the pyroptosis of granulosa cells in ovary, and these results were consistent with the results predicted in network pharmacology pathway analysis.

Frankly speaking, BCR is an empirical prescription that has been derived from decades of clinical TCM practice. Based on its clinical effects in the treatment of female infertility, BCR was developed into a marketed product, tradename Yueliang Yin, as a functional food in China. We are aware that there is a limitation in clinical evidence of the efficacy of BCR in the treatment of female infertility due to earlier TCM practices failing to follow a design that meets the standards of evidence-based medicine. To construct scientific evidence for the effects of BCR in the treatment of female infertility, a clinical trial of BCR is being undertaken.

**Figure 9:**
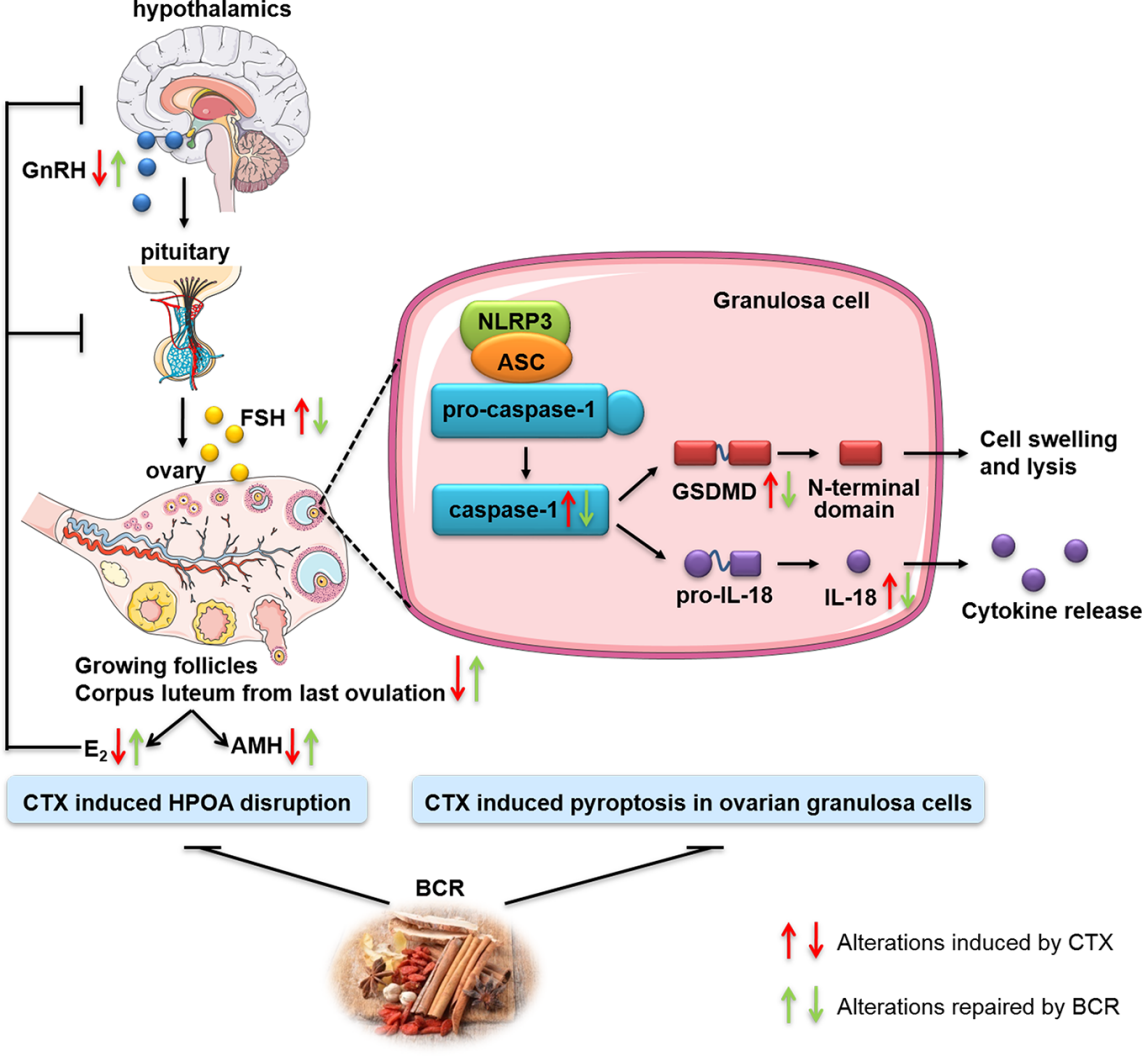
Schematic illustration of the possible mechanism of DOR treated by BCR.

Generally, we demonstrated that BCR, an ancient Chinese herbal decoction, had protective effects against CTX damage on ovary. DOR rats showed a prolonged estrous cycle, decreased ovarian index, decreased number of growing follicles, and alterations in hormone levels, including increased FSH and decreased AMH, E_2_, and GnRH. Interestingly, these changes were significantly improved after BCR treatments, especially high dosage, indicating that the Chinese medicine BCR might represent a positive therapy for DOR. For the possible action mechanisms of BCR on DOR, it was summarized in **Figure 9**.

## Conclusions

The chemical compositions of BCR were identified in the present study. BCR was demonstrated to show protective effects on DOR. The possible mechanisms of BCR in DOR might be mediated by regulating gonadal hormones of the hypothalamic-pituitary-ovarian axis (HPOA) and protecting granulosa cells in ovary against pyroptosis.

## Data Availability Statement

All datasets generated for this study are included in the article/supplementary material.

## Ethics Statement

The animal study was reviewed and approved by Ethics Committee for Animal Care and Treatment of Beijing University of Chinese Medicine.

## Author Contributions

MJ, WW, and JZ performed the experiment and analyzed the data. CW, SY, YB, JL, and PL performed the BCR extraction and analysis. MJ and WW drafted the manuscript. Y-TX and TW revised the manuscript.

## Funding

This work was supported by Chongqing JuqiNuomei Pharmaceutical Co., Ltd. (Chongqing, China) (No. 3010071720022).

## Conflict of Interest

The authors declare that the research was conducted in the absence of any commercial or financial relationships that could be construed as a potential conflict of interest.
